# Mitochondrial Redox Failure Links Senescence-like Foam Cell Stress to Ferroptosis and Plaque Non-Resolution in Atherosclerosis

**DOI:** 10.3390/antiox15091061

**Published:** 2026-08-25

**Authors:** Phyu Phyu Khin, Hla Myat Mo Mo, Cuk-Seong Kim

**Affiliations:** Department of Physiology & Medical Science, College of Medicine, Chungnam National University, Daejeon 35015, Republic of Korea; phyuphyukim92@gmail.com (P.P.K.); nicholelynn7@gmail.com (H.M.M.M.)

**Keywords:** atherosclerosis, foam cell, vascular smooth muscle cell, mitochondrial redox failure, senescence-like foam cell, redox-threshold model, ferroptosis, GPX4, efferocytosis, plaque non-resolution, necrotic core

## Abstract

Foam cells are central to atherosclerotic plaque development, but their pathological significance in advanced lesions extends beyond lipid accumulation. Under chronic exposure to oxidized lipoproteins, cholesterol crystals, inflammatory cytokines, hypoxia, lysosomal stress, and mitochondrial injury, lipid-loaded foam cells of macrophage and vascular smooth muscle cell (VSMC) origin may acquire maladaptive stress phenotypes. In this focused review, we propose a redox-threshold model, defined as the transition point at which mitochondrial antioxidant and metabolic buffering capacity is exceeded, allowing lipid peroxide accumulation to shift senescence-like foam cells toward ferroptosis susceptibility and defective plaque resolution. Progressive mitochondrial reactive oxygen species (ROS) accumulation, impaired NADPH-dependent antioxidant buffering, defective glutathione and thioredoxin recycling, mitophagy impairment, and reduced GPX4-mediated lipid peroxide detoxification may converge to promote iron-dependent lipid peroxidation. If lipid-peroxidized or dying foam cells are not efficiently cleared, oxidized lipids, cellular debris, and inflammatory signals accumulate, promoting secondary necrosis, necrotic core expansion, plaque non-resolution, and instability. We explicitly distinguish established processes from mechanistically supported inferences and hypothesis-generating links, emphasizing that the complete senescence-like stress-to-ferroptosis-to-non-resolution sequence remains a testable framework rather than an established linear pathway. This focused framework suggests that advanced plaque stabilization may require strategies that preserve mitochondrial redox resilience, limit ferroptotic lipid peroxidation, and enhance efferocytosis-mediated resolution.

## 1. Introduction: Foam Cell Fate Failure in Advanced Atherosclerosis

Atherosclerosis is a chronic inflammatory and metabolic disease of the arterial wall and remains a major cause of myocardial infarction, ischemic stroke, and peripheral artery disease [1,2,3]. Lipid-lowering therapy has substantially reduced cardiovascular events, yet residual cardiovascular risk persists in many patients, particularly those with advanced plaque burden, diabetes, chronic kidney disease, aging-related inflammation, or persistent inflammatory stress [4,5,6,7,8,9]. These observations indicate that plaque progression and instability are not determined solely by circulating lipid levels. In advanced lesions, oxidative stress, defective inflammation resolution, regulated cell death, necrotic core expansion, and fibrous cap destabilization become major determinants of clinical risk.

Foam cells are a defining feature of atherosclerotic plaques. They are classically described as lipid-laden macrophages generated by excessive uptake of modified lipoproteins and insufficient cholesterol efflux [10,11,12]. In this model, apoB-containing lipoproteins enter the arterial intima, undergo retention and oxidative or enzymatic modification, and are taken up through scavenger receptor-mediated pathways [12,13,14]. When lipid uptake exceeds the capacity for lipid processing and efflux, cells acquire a foamy phenotype. However, advanced plaque foam cells are not exclusively macrophage-derived. Vascular smooth muscle cells (VSMCs) undergo extensive phenotypic modulation and contribute substantially to plaque foam-cell populations. Mouse lineage-tracing studies demonstrate multiple SMC-derived plaque states, including macrophage-like populations [15], whereas human coronary histology indicates a substantial contribution of SMC-derived cells to the foam-cell pool [16]. Single-cell studies further reveal diverse phenotypically modulated SMC states in mouse and human plaques [17,18,19].

Accordingly, the proposed redox-threshold model should be interpreted in a lineage-aware manner. Macrophage-derived and VSMC-derived foam cells may share lipid accumulation and redox stress, but their senescence-like programs, ferroptosis susceptibility, efferocytosis-related functions, and contributions to plaque stability may differ. This review therefore focuses on foam cell fate failure while acknowledging that foam cells represent lineage-diverse stress states rather than a macrophage-only population. Human single-cell analyses also identify heterogeneous foamy macrophage states, further supporting a state-based rather than binary view of plaque macrophages [20].

Experimental atherosclerosis studies have identified intimal foam cells expressing senescence-associated markers, and transgenic or pharmacological elimination of senescent cells in Ldlr-deficient mice reduced lesion growth and features of plaque instability [21]. General cellular senescence is characterized by persistent stress signaling, DNA damage responses, mitochondrial dysfunction, altered metabolism, and production of the senescence-associated secretory phenotype (SASP) [22,23,24,25,26,27]. However, whether macrophage- or VSMC-derived foam cells fully satisfy canonical senescence criteria in human plaques remains unresolved. Accordingly, this review uses the term “senescence-like foam cell” to describe a persistent damage-associated stress state rather than mechanistic equivalence to classical replicative senescence. These cells may remain viable but dysfunctional and act as persistent inflammatory hubs within the plaque. Their pathological significance may extend beyond SASP production: senescence-like stress may represent a vulnerable pre-death state in which foam cells progressively lose adaptive capacity. Mitochondria are well positioned to regulate this transition because they control ROS generation, nicotinamide adenine dinucleotide phosphate (NADPH)-dependent antioxidant buffering, glutathione and thioredoxin redox systems, mitophagy, inflammasome activation, and cell death susceptibility [28,29,30]. When mitochondrial redox buffering remains sufficient, senescence-like foam cells may tolerate lipid and inflammatory stress; when mitochondrial redox homeostasis fails, they may become vulnerable to lipid peroxide-driven injury.

Ferroptosis is an iron-dependent form of regulated cell death driven by excessive lipid peroxidation [31,32,33]. Foam cells may be particularly susceptible to ferroptotic stress because they contain abundant oxidizable lipids, are exposed to oxidized lipoproteins and redox-active iron, and depend on glutathione peroxidase 4 (GPX4)-mediated detoxification of phospholipid hydroperoxides for survival [31,32,33,34,35,36]. In senescence-like foam cells, progressive mitochondrial reactive oxygen species accumulation, impaired NADPH-dependent antioxidant recycling, defective glutathione and thioredoxin systems, mitophagy impairment, and reduced GPX4 activity may lower the threshold for ferroptotic injury. The plaque-level consequence of this transition depends on efferocytosis. Efficient efferocytosis removes dying cells, suppresses inflammation, prevents secondary necrosis, and promotes tissue resolution [37,38,39,40,41,42,43]. In advanced plaques, however, efferocytosis becomes defective through impaired Myeloid-epithelial-reproductive tyrosine kinase (MerTK) and Low-density lipoprotein receptor (LRP1) signaling, increased Cluster of Differentiation 47—Signal regulatory protein α (CD47-SIRPα) ‘don’t eat me’ signaling, oxidative stress, proteolytic activity, cholesterol overload, and macrophage metabolic dysfunction. When lipid-peroxidized or dying foam cells are not efficiently cleared, oxidized lipids, cellular debris, mitochondrial components, and inflammatory mediators accumulate, promoting secondary necrosis, necrotic core expansion, plaque non-resolution, and instability.

**Figure 1:**
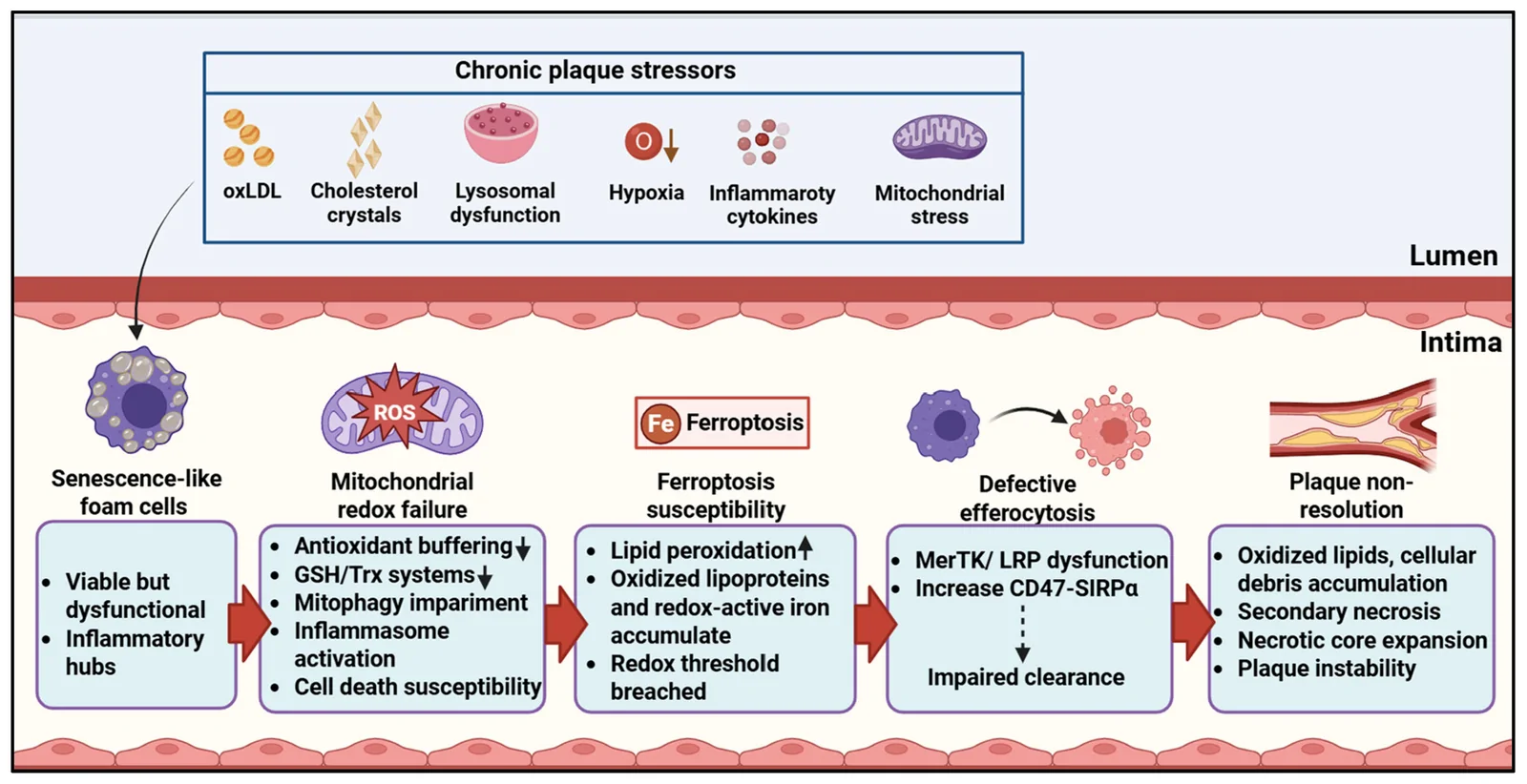
Redox threshold model linking senescence-like foam cell stress to ferroptosis and plaque non-resolution. Chronic plaque stressors may drive lineage-diverse foam cells into a senescence-like inflammatory state. Senescence-like foam cells can persist as SASP-producing inflammatory hubs while mitochondrial redox buffering remains sufficient. Progressive mitochondrial ROS accumulation, impaired NADPH-dependent antioxidant recycling, defective glutathione and thioredoxin systems, mitophagy impairment, and reduced GPX4-mediated lipid peroxide detoxification may lower the redox threshold for ferroptotic injury. If lipid-peroxidized or dying foam cells are not efficiently cleared by efferocytosis, oxidized lipids, cellular debris, and inflammatory material accumulate, leading to secondary necrosis, necrotic core expansion, plaque non-resolution, and plaque instability. In the schematic, black arrows indicate the initial effects of chronic plaque stressors and foam cell formation stage, respectively, while red arrows indicate the subsequent progression from senescence-like foam cells to plaque formation. Created in BioRender. Kim, C. S. (2026) https://BioRender.com/3pdcjxc.

In this focused review, we propose that mitochondrial redox failure lowers the threshold at which senescence-like foam cells become ferroptosis-prone, and that defective efferocytosis converts this cell fate failure into plaque non-resolution. This review is deliberately restricted to the sequence linking senescence-like foam cell stress, mitochondrial redox failure, ferroptosis susceptibility, and defective efferocytosis. It does not attempt to cover all mechanisms of atherosclerosis progression, all macrophage phenotypes, or all cardiovascular therapies. This narrower scope is intended to provide a testable framework for understanding advanced plaque non-resolution. We summarize this concept as a redox threshold model in which senescence-like foam cells remain viable while mitochondrial redox buffering and GPX4-mediated lipid peroxide detoxification are preserved, but become ferroptosis-prone when these protective systems fail (Figure 1).

To avoid overinterpreting the proposed sequence, this review distinguishes three levels of evidence. First, several individual processes are well established or strongly supported, including foam cell formation, senescence/SASP signaling, mitochondrial oxidative stress, ferroptosis-related lipid peroxidation, and defective efferocytosis in advanced atherosclerosis. Second, some relationships are mechanistically supported but incompletely proven within the same foam cell population, such as the link between mitochondrial redox failure and increased ferroptosis susceptibility. Third, the full sequence connecting senescence-like foam cell stress, mitochondrial redox failure, ferroptosis susceptibility, defective efferocytosis, and plaque non-resolution is presented as a hypothesis-generating redox-threshold model rather than as an established linear pathway. Here, the redox-threshold model refers to the point at which mitochondrial antioxidant buffering and GPX4-dependent lipid peroxide detoxification are no longer sufficient to restrain lipid peroxidation, thereby increasing ferroptosis susceptibility and the risk of plaque non-resolution.

## 2. Senescence-like Foam Cells and Mitochondrial Redox Vulnerability

Senescence-like foam cell stress can be viewed as a maladaptive state linking chronic plaque injury to mitochondrial redox vulnerability. In early lesions, macrophage-derived foam cells may retain adaptive functions, including lipid storage, cholesterol efflux, antioxidant buffering, and efferocytosis. In advanced plaques, both macrophage-derived and VSMC-derived foam cells may be exposed to persistent oxidized lipoproteins, cholesterol crystals, inflammatory cytokines, hypoxia, lysosomal stress, defective autophagy, and mitochondrial dysfunction. These stressors may not immediately induce cell death; instead, they may generate lineage-specific senescence-like stress states that persist within the plaque microenvironment [15,16,18,19,21,22,23,24,25,26,27].

### 2.1. Senescence-like Foam Cell Stress as a Persistent Inflammatory State

Key features of classical cellular senescence include sustained stress signaling, DNA damage, metabolic reprogramming, mitochondrial dysfunction, and SASP secretion [21,22,23,24,25,26,27]. However, the term senescence should be applied carefully to foam cells. Macrophages do not undergo classical replicative senescence in the same way as proliferating fibroblasts or epithelial cells, and VSMC-derived foam cells may follow distinct phenotypic trajectories after transdifferentiation. Therefore, we use the term “senescence-like foam cell” to denote a persistent damage-associated stress state rather than a direct equivalent of replicative senescence.

A senescence-like foam cell state should not be defined by inflammatory cytokine expression alone, because IL-1beta, TNF-alpha, and other SASP-like mediators overlap with inflammatory macrophage activation. Operationally, this state should require a composite signature including lineage identity, lipid loading, p16/p21 or p53 pathway activation, DNA damage signaling, mitochondrial dysfunction, impaired autophagy or mitophagy, persistent SASP-like output, reduced adaptive capacity, and spatial persistence within advanced plaques. Inflammatory macrophage activation is itself heterogeneous and should be defined using the cellular source, activating stimulus, and combinations of markers rather than a rigid M1/M2 binary classification [44]. Thus, expression of IL1B, TNF, NOS2, CD80/CD86, or related inflammatory programs alone is insufficient to establish a senescence-like foam cell state.

### 2.2. Plaque Stressors That Reinforce Senescence-like Foam Cell Stress

Several plaque-associated stressors can reinforce senescence-like foam cell programming. Modified lipoproteins and oxidized lipid species increase intracellular lipid burden and oxidative stress. Cholesterol crystals and lysosomal dysfunction activate inflammatory signaling and impair cellular repair capacity. Hypoxia limits metabolic flexibility and promotes mitochondrial stress. Inflammatory cytokines can further sustain macrophage activation and prevent resolution [45,46,47,48,49,50]. Defective autophagy also contributes to this process. Autophagy supports lipid droplet turnover, lysosomal homeostasis, damaged organelle clearance, and stress adaptation. When autophagic flux is impaired, lipid droplets, damaged mitochondria, oxidized proteins, and dysfunctional lysosomes accumulate [48,49]. This creates a self-reinforcing loop in which lipid stress promotes organelle dysfunction, increases oxidative stress, and amplifies inflammatory signaling. Mitochondrial dysfunction is particularly important because it links senescence-like stress to redox failure. Cells undergoing canonical senescence frequently exhibit mitochondrial dysfunction and increased ROS production [27,51,52], whereas alterations in mitochondrial dynamics and quality-control pathways may further contribute to cellular stress [53]. In foam cells, lipid overload and defective cholesterol trafficking can impair mitochondrial respiration and increase mitochondrial ROS production [28,29,30]. This mitochondrial stress may reinforce DNA damage responses, inflammasome activation, nuclear factor kappa-light-chain-enhancer of activated B cells (NF-kappaB) signaling, and SASP production. Therefore, mitochondrial dysfunction may not simply be a consequence of senescence-like foam cell stress; it may actively maintain and amplify the senescence-like phenotype in advanced plaques.

### 2.3. Mitochondrial Redox Buffering as a Survival Checkpoint

Senescence-like foam cells persist in a plaque environment rich in oxidized lipids, inflammatory cytokines, hypoxia, iron, and reactive oxygen species. Under these conditions, survival depends on mitochondrial redox buffering. Mitochondria regulate ROS generation, antioxidant defense, mitophagy, inflammatory signaling, and cell death susceptibility [28,29,30]. When these systems remain coordinated, foam cells may tolerate lipid overload and inflammatory stress. When mitochondrial redox buffering fails, however, foam cells become vulnerable to lipid peroxide-driven injury. NADPH-dependent antioxidant buffering is central to this process. Because NADPH supports glutathione and thioredoxin recycling and thereby sustains peroxide-detoxifying capacity [54,55,56,57], reduced NADPH availability would be expected to weaken redox buffering in chronically stressed foam cells. The glutathione and thioredoxin systems function together as a redox defense network. Reduced glutathione supports glutathione peroxidase activity, including GPX4-mediated detoxification of phospholipid hydroperoxides [34,58,59,60]. Thioredoxin and thioredoxin reductase systems maintain protein redox balance and support peroxide detoxification [59]. In the proposed framework, these established biochemical mechanisms provide a plausible basis for redox vulnerability in lipid-loaded foam cells, which are continuously exposed to oxidized lipoproteins, mitochondrial ROS, and inflammatory mediators.

### 2.4. From Mitochondrial Dysfunction to Redox Vulnerability

The transition from senescence-like foam cell persistence to redox vulnerability occurs when mitochondrial ROS generation exceeds antioxidant and repair capacity. Persistent mitochondrial ROS can reinforce SASP production, inflammasome activation, DNA damage signaling, and lipid peroxide formation. At the same time, impaired NADPH-dependent recycling weakens glutathione and thioredoxin systems, reducing the ability of foam cells to maintain GPX4-dependent lipid peroxide detoxification [54,55,56,57]. Mitophagy failure further amplifies this vulnerability. Damaged mitochondria are major sources of ROS and mitochondrial danger signals. Efficient mitophagy removes dysfunctional mitochondria and limits persistent oxidative stress. When mitophagy is impaired, damaged mitochondria accumulate, mitochondrial ROS increases, and redox stress becomes self-sustaining [51,52,53]. In senescence-like foam cells, defective mitophagy may therefore reinforce both inflammatory persistence and susceptibility to lipid peroxide-driven injury.

**Figure 2:**
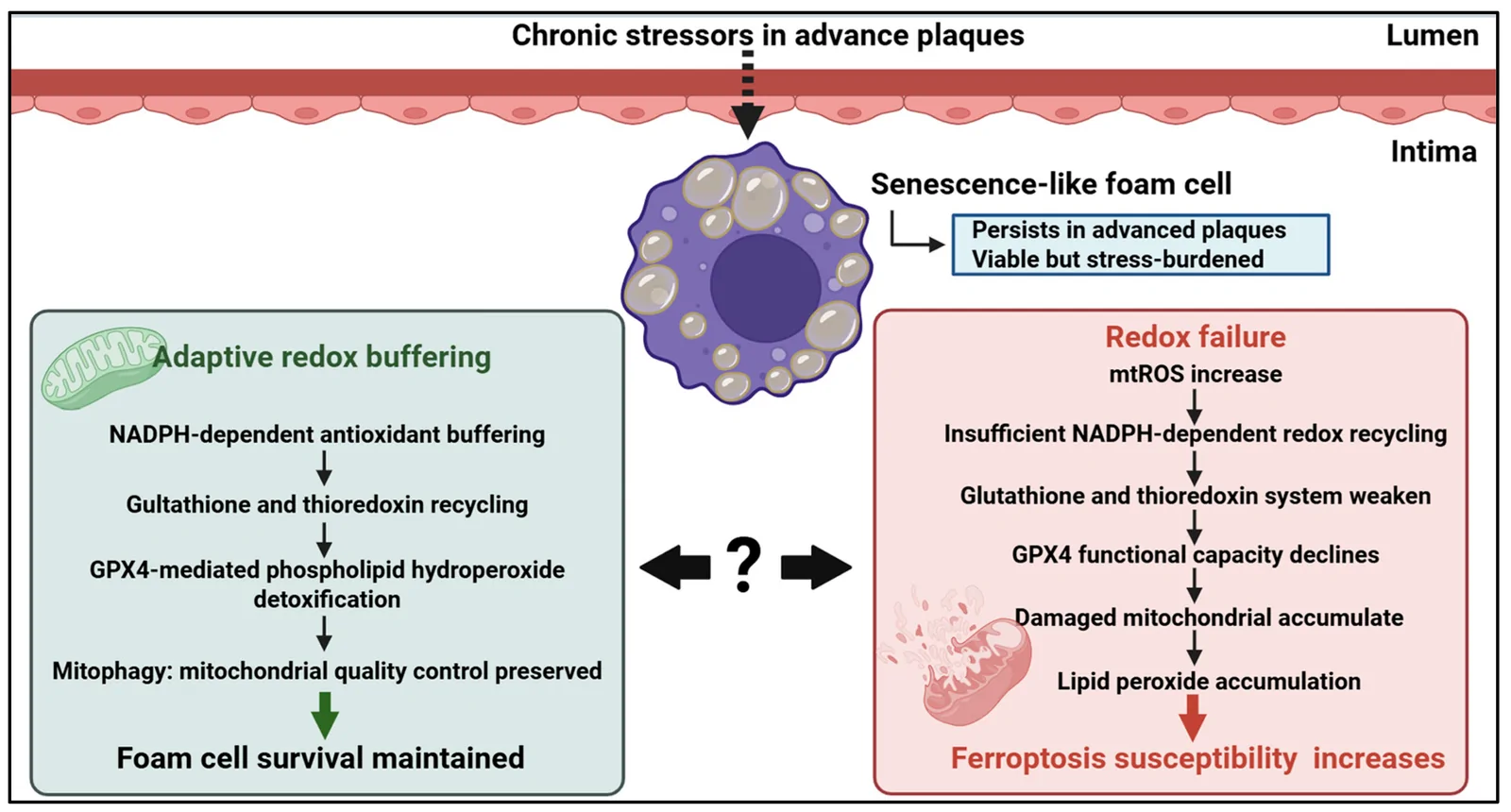
Mitochondrial redox vulnerability in senescence-like foam cells. Senescence-like foam cells persist in advanced plaques under chronic exposure to oxidized lipids, inflammatory cytokines, cholesterol crystals, hypoxia, lysosomal stress, and mitochondrial injury. Mitochondria act as redox sensors and survival checkpoints in this setting. Under adaptive conditions, NADPH-dependent antioxidant buffering supports glutathione and thioredoxin recycling, maintains GPX4-mediated phospholipid hydroperoxide detoxification, and preserves mitochondrial quality control through mitophagy. When mitochondrial ROS production increases and NADPH-dependent redox recycling becomes insufficient, glutathione and thioredoxin systems weaken, GPX4 functional capacity declines, and damaged mitochondria accumulate. This redox imbalance lowers the threshold for lipid peroxide accumulation and increases ferroptosis susceptibility. Abbreviations: ROS, reactive oxygen species; mtROS, mitochondrial ROS; NADPH, reduced nicotinamide adenine dinucleotide phosphate; GPX4, glutathione peroxidase 4. In the schematic, the black dashed arrow indicates the effect of chronic plaques stressor and whereas the black arrows in each panel box indicate the subsequent transition foam cell stage. The green panel and green arrow represent adaptive redox buffering and maintained foam cell survival, respectively. The red panel and red arrow represent redox failure and increased susceptibility to ferroptosis, respectively. The question mark (?) and black arrows indicates foam cell fate may shift toward either survival or ferroptosis depending on macrophage redox stage. Created in BioRender. Kim, C. S. (2026) https://BioRender.com/0yr9etf.

This section establishes the key intermediate state in the proposed model: senescence-like foam cells are viable but redox-vulnerable. They can remain within plaques as SASP-producing inflammatory cells while mitochondrial redox buffering remains sufficient. However, continued mitochondrial ROS production, impaired NADPH-dependent antioxidant recycling, weakened glutathione and thioredoxin systems, defective mitophagy, and declining GPX4 functional capacity may lower the threshold for ferroptotic injury. The major mitochondrial redox systems that determine whether senescence-like foam cells remain viable or progress toward lipid peroxide-driven injury are summarized in Figure 2.

## 3. Ferroptosis Susceptibility and the Redox Threshold Model

Ferroptosis susceptibility represents a potential downstream consequence of mitochondrial redox vulnerability in senescence-like foam cells. Ferroptosis is an iron-dependent form of regulated cell death driven by excessive lipid peroxidation and failure of lipid peroxide detoxification [31,32,33,35,36,61,62]. General ferroptosis studies establish central roles for GPX4, glutathione, ACSL4-dependent phospholipid remodeling, and iron-dependent lipid radical propagation [31,32,33,34,35,36,61,62,63,64,65,66]. In atherosclerosis-related models, emerging preclinical evidence further indicates that macrophage and foam-cell ferroptosis can be modulated through autophagy-NRF2 and KEAP1-NRF2-GPX4/xCT pathways [67,68]. However, direct evidence that senescence-like foam cells undergo ferroptosis in human plaques remains limited. Lineage-diverse foam cells may nevertheless be particularly prone to ferroptotic stress because they contain abundant oxidizable lipids and reside in a microenvironment enriched with oxidized lipoproteins, inflammatory oxidants, redox-active iron, mitochondrial stress, and defective clearance. Thus, ferroptosis susceptibility may provide a mechanistically plausible link between senescence-like foam cell persistence and plaque non-resolution.

A major caveat is that ferroptosis cannot be assigned in complex plaques using a single marker. Lipid peroxidation markers such as 4-hydroxynonenal (4-HNE), malondialdehyde (MDA), or oxidized phospholipids may also accumulate during secondary necrosis, intraplaque hemorrhage, or generalized oxidative stress. Therefore, ferroptosis-like injury in advanced plaques should be interpreted using convergent evidence, including preservation of cell identity, spatial localization, phospholipid hydroperoxide accumulation, altered GPX4/SLC7A11/ACSL4/FSP1-related pathways, iron-dependent injury signatures, supportive ultrastructural changes while recognizing that mitochondrial morphology alone is not diagnostic, and, where possible, rescue by ferroptosis-targeted interventions [61,62,63].

### 3.1. Lipid Peroxidation as a Central Stress in Foam Cells

Foam cells are defined by intracellular lipid accumulation, but lipid storage and lipid peroxidation have different pathological implications [69]. In early or adaptive foam cell states, lipid droplets may help buffer excess cholesterol and fatty acids, reducing immediate lipotoxicity. However, in advanced plaques, persistent oxidative stress, mitochondrial ROS, oxidized lipoproteins, and inflammatory signaling can convert lipid accumulation into lipid peroxide stress. Polyunsaturated fatty acid (PUFA)-containing phospholipids are especially vulnerable to peroxidation. Once phospholipid hydroperoxides accumulate, membrane integrity becomes increasingly unstable, and lipid radical propagation can amplify oxidative damage. This process is particularly relevant in foam cells because they are exposed to both intracellular sources of oxidants and extracellular oxidized lipid species. Thus, foam cell fate is not determined simply by the amount of lipid stored, but by the balance between oxidizable lipid burden and lipid peroxide detoxification capacity [31,32,33,35,36,61,62,63,64,65,70].

### 3.2. GPX4-Dependent Lipid Peroxide Detoxification

GPX4 is a central suppressor of ferroptosis because it detoxifies phospholipid hydroperoxides and prevents lipid peroxide-driven membrane damage [31,32,33,34]. In senescence-like foam cells, GPX4 function may be especially important because these cells are chronically exposed to mitochondrial ROS, oxidized lipoproteins, inflammatory cytokines, and lysosomal stress. As long as GPX4-dependent lipid peroxide detoxification is preserved, lipid peroxidation may remain controlled and compatible with cell survival. However, GPX4 activity depends on reduced glutathione, and glutathione recycling depends on NADPH-supported redox systems [34,54,55,56,57,58,59,60]. Therefore, GPX4 failure should not be viewed only as reduced GPX4 expression or direct enzymatic inhibition. It can also occur functionally when NADPH availability declines, glutathione becomes depleted, thioredoxin-dependent redox support weakens, or mitochondrial ROS production overwhelms detoxification capacity. In this sense, ferroptosis susceptibility reflects a network failure of the NADPH-glutathione/thioredoxin-GPX4 axis rather than a single molecular defect.

### 3.3. Oxidizable Lipids, Acyl-CoA Synthetase Long-Chain Family Member 4 (ACSL4) and Iron-Dependent Injury

The sensitivity of foam cells to ferroptosis also depends on membrane lipid composition. ACSL4 promotes incorporation of polyunsaturated fatty acids into phospholipids and thereby increases the pool of oxidizable membrane lipids [36]. In plaques, oxidized phospholipids and lipid peroxidation products may accumulate in macrophage-rich regions, particularly near necrotic cores, hemorrhagic areas, and sites of intense inflammation. These lipid species may further amplify macrophage stress and ferroptosis susceptibility. Iron is another important determinant of lipid peroxide propagation. Redox-active iron can promote lipid radical formation and accelerate oxidative membrane injury. In advanced plaques, iron may derive from intraplaque hemorrhage, erythrocyte degradation, heme metabolism, ferritin turnover, or macrophage iron handling. When iron-dependent radical formation converges with mitochondrial ROS and insufficient GPX4 activity, senescence-like foam cells may become increasingly vulnerable to ferroptotic injury [61,62,63,65,70]. Nevertheless, ferroptosis in atherosclerosis should be interpreted cautiously. Advanced plaques contain multiple forms of cell stress and cell death, including apoptosis, necroptosis, pyroptosis, and secondary necrosis. Ferroptosis-related markers may coexist with other death pathways. Therefore, ferroptosis should be described as a plausible and testable component of foam cell fate failure, rather than assumed to be the dominant mechanism in all advanced plaques.

### 3.4. The Redox Threshold Model

The redox threshold model proposes that senescence-like foam cells remain viable while mitochondrial redox buffering and lipid peroxide detoxification are sufficient, but become increasingly ferroptosis-prone as these protective systems fall below a critical functional threshold [30,32,66,71]. The balance between lipid peroxide generation and lipid peroxide detoxification determines the threshold. On the injury side, mitochondrial ROS, oxidized lipoproteins, inflammatory oxidants, redox-active iron, lysosomal dysfunction, and oxidizable phospholipids increase lipid peroxide burden. On the protective side, NADPH-dependent antioxidant buffering, glutathione and thioredoxin recycling, GPX4 activity, and mitophagy-dependent mitochondrial quality control restrain lipid peroxide accumulation [28,29,30,31,32,33,34,35,36,54,55,56,57,58,59,60,61,62,63,64,65,70]. In the proposed model, a senescence-like foam cell would become increasingly ferroptosis-prone when lipid-peroxide-generating pressure exceeds the capacity of these protective systems. This threshold should not be viewed as a simple on-off switch; it is more appropriately understood as a systems-level failure. Progressive mitochondrial ROS accumulation can increase lipid peroxide formation. NADPH insufficiency can impair glutathione and thioredoxin recycling. Reduced glutathione availability can weaken GPX4 function. Defective mitophagy can allow damaged mitochondria to persist as ROS-generating organelles. Iron and oxidizable phospholipids can accelerate lipid radical propagation. Together, these changes may lower the redox threshold for ferroptotic injury [28,29,30,32,36,65].

### 3.5. From Senescence-like Foam Cell Stress to Ferroptosis Susceptibility

The proposed transition from senescence-like foam cell stress to ferroptosis susceptibility does not imply that all senescence-like foam cells inevitably die by ferroptosis. Rather, we hypothesize that a senescence-like stress state may create a cellular background in which progressive mitochondrial redox failure increases susceptibility to ferroptotic injury. Senescence-like foam cells may persist as SASP-producing inflammatory cells for an extended period. During this phase, they remain viable but may experience sustained mitochondrial dysfunction, oxidative stress, impaired repair capacity, and reduced metabolic flexibility [27,51,52]. As redox buffering declines, these cells may gradually lose the ability to detoxify lipid peroxides. At this point, lipid peroxidation may shift from a stress signal to a lethal process. Phospholipid hydroperoxides can accumulate, membrane damage can increase, and iron-dependent lipid radical propagation may become more difficult to restrain. This creates a ferroptosis-prone foam cell state positioned conceptually between senescence-like persistence and irreversible lipid-peroxide-driven injury [32,36,65]. This hypothesis provides a mechanistic framework linking chronic inflammatory plaque stress to necrotic plaque progression without requiring a single abrupt event. Senescence-like foam cells may first sustain inflammation through SASP signaling, then become redox-vulnerable, and finally develop ferroptosis susceptibility when lipid peroxide detoxification fails. This proposed transition is gradual, context-dependent, and potentially reversible if mitochondrial redox balance or GPX4-mediated lipid peroxide clearance is restored before irreversible membrane damage occurs [27,32,51,63].

### 3.6. Experimental Implications

The redox threshold model generates several experimentally testable predictions. Senescence-like foam cells located near necrotic cores, hypoxic regions, or hemorrhagic plaque areas should show stronger mitochondrial dysfunction, lipid peroxidation, and ferroptosis-related signatures than adaptive foam cells. Foam cells with impaired NADPH-glutathione/thioredoxin-GPX4 buffering should be more sensitive to ferroptosis-like injury. Conversely, restoring mitochondrial redox buffering, enhancing mitophagy, preserving GPX4 activity, limiting iron-dependent lipid peroxidation, or trapping lipid radicals should reduce ferroptosis susceptibility [31,32,33,34,35,36,54,55,56,57,58,59,60,61,62,63,64,65,70]. Validation will require approaches that preserve cell identity and spatial context. Single-cell transcriptomics, spatial transcriptomics, lipidomics, redox imaging, mitochondrial reporters, and functional rescue experiments will be needed to determine whether senescence-like foam cells directly acquire ferroptosis-prone features in vivo [17,19,72,73,74,75,76,77]. Until such evidence is available, the senescence-like stress-to-ferroptosis transition should be presented as a plausible framework rather than a fully established pathway. Together, these concepts define mitochondrial redox failure as the central event that lowers the ferroptotic threshold in senescence-like foam cells. The next section examines how defective efferocytosis converts this cell-intrinsic vulnerability into plaque non-resolution, necrotic core expansion, and instability.

In advanced human plaques, primary ferroptosis should also be distinguished from secondary necrosis or nonspecific oxidative damage. A rigorous designation of ferroptosis-like injury should require multi-domain evidence rather than isolated staining for lipid peroxidation. Such evidence may include preserved cell identity, spatial localization outside acellular necrotic debris, phospholipid hydroperoxide accumulation, altered GPX4/SLC7A11/ACSL4/FSP1 signaling, iron-dependent injury signatures, compatible mitochondrial morphology, and functional rescue by ferroptosis-targeted interventions in experimental models.

## 4. Defective Efferocytosis and Therapeutic Opportunities

Mitochondrial redox failure and ferroptosis susceptibility may explain how senescence-like foam cells become vulnerable to lipid peroxide-driven injury. However, the plaque-level consequence of this injury depends largely on whether damaged or dying cells are efficiently cleared. Efferocytosis is the process by which phagocytes recognize, engulf, and remove dying cells. In atherosclerosis, efficient efferocytosis limits inflammation, prevents secondary necrosis, and promotes tissue resolution [37,38,39,40,41,42,43,78,79]. When efferocytosis fails, foam cell death is converted into extracellular debris, necrotic core expansion, persistent inflammation, and plaque non-resolution.

### 4.1. Efferocytosis as a Resolution Checkpoint

In early atherosclerotic lesions, efferocytosis can limit the inflammatory consequences of macrophage apoptosis or cellular stress. Dying cells expose ‘eat me’ signals that are recognized by phagocytic receptors and bridging molecules, allowing macrophages to engulf and process cellular debris. This process is not simply waste removal. It also promotes anti-inflammatory signaling, lipid handling, and tissue repair responses [37,38,39,40,41,42,43]. In advanced plaques, however, efferocytosis becomes progressively defective. Several mechanisms contribute to this defect. MerTK signaling can be impaired or disrupted by proteolytic cleavage in inflammatory plaque environments. LRP1-dependent clearance pathways may be weakened. Increased CD47-SIRPα signaling can suppress phagocytic engulfment by providing a ‘don’t eat me’ signal. In addition, cholesterol overload, lysosomal dysfunction, oxidative stress, and mitochondrial injury can reduce the ability of macrophages to engulf and metabolically process dying cells [40,41,42,43]. This clearance defect is particularly important in the context of ferroptosis-prone foam cells. Ferroptotic or severely lipid-peroxidized foam cells may generate oxidized phospholipids, lipid aldehydes, damaged membrane fragments, mitochondrial components, and inflammatory debris. If these materials are not efficiently cleared, they may amplify oxidative stress and inflammatory signaling in neighboring cells. Thus, defective efferocytosis transforms foam cell injury from a cell-intrinsic event into a tissue-level driver of plaque non-resolution [35,39,42,43,80].

### 4.2. Recognition and Clearance of Ferroptotic Foam Cells: A Resolution Paradox

Ferroptotic foam cell clearance raises an important biological paradox. Unlike apoptosis, ferroptosis is driven by lipid peroxidation and can generate oxidized phospholipids, lipid aldehydes, mitochondrial fragments, and pro-inflammatory debris. Therefore, ferroptotic foam cells may not be equivalent to apoptotic cells as efferocytic targets. Some oxidized phospholipids may function as recognition signals; for example, oxygenated phosphatidylethanolamine species have been reported to act as macrophage-recognized signals through TLR2-dependent mechanisms [80]. However, whether this pathway operates efficiently in advanced atherosclerotic plaques remains uncertain.

In advanced plaques, recognition and clearance of ferroptotic foam cells may be compromised at multiple levels. MerTK-dependent efferocytosis can be impaired by proteolytic cleavage, LRP1-mediated clearance may be weakened, and increased CD47-SIRPα signaling may suppress engulfment. In addition, macrophages exposed to cholesterol overload, oxidized lipids, hypoxia, and mitochondrial redox stress may have reduced capacity to process lipid-peroxidized cargo after engulfment. Thus, ferroptotic cells may be recognizable but not efficiently cleared or safely metabolized in the advanced plaque microenvironment.

A further, largely untested possibility is that successful clearance of ferroptotic cells requires sufficient post-engulfment redox and metabolic capacity in the phagocyte. Potential protective mechanisms, inferred from established macrophage lipid-handling, efferocytosis, and ferroptosis biology, may include lysosomal processing, lipid sequestration and efflux, NRF2-dependent antioxidant responses, glutathione and thioredoxin recycling, GPX4 activity, and mitochondrial quality control [39,62,79,81]. Whether these mechanisms are sufficient to prevent secondary lipid-peroxide injury after uptake of ferroptotic foam-cell debris remains to be directly established. Thus, defective efferocytosis in advanced plaques may involve not only corpse recognition and engulfment but also inadequate post-engulfment processing of oxidized cargo.

### 4.3. From Uncleared Foam Cells to Plaque Non-Resolution

Plaque non-resolution reflects a failure to remove inflammatory and necrotic material from the lesion. When damaged foam cells are not cleared, they may undergo secondary necrosis or persist as fragmented debris, contributing to extracellular lipid accumulation, cholesterol crystal deposition, necrotic core expansion, and sustained inflammatory signaling [37,78,79]. The necrotic core then becomes a local source of danger-associated molecular patterns, oxidized lipids, proteases, and matrix-destabilizing signals. Within the proposed framework, chronic plaque stress may promote senescence-like foam-cell states; progressive redox failure may increase ferroptosis susceptibility; and, when clearance is inadequate, the resulting cellular injury may contribute to necrotic core expansion and plaque non-resolution. These events should be viewed as mechanistically connected possibilities rather than as a temporally proven linear cascade. The key problem is therefore not simply the presence of foam cells, but failure to maintain redox resilience, avoid lipid peroxide-driven injury, and achieve efficient clearance when damage occurs. In this sense, advanced atherosclerosis may be viewed as a disease of lipid accumulation, redox failure, and failed resolution.

### 4.4. Therapeutic Strategies Targeting Foam Cell Fate Failure

Reducing atherogenic lipoprotein burden remains the foundation of atherosclerosis prevention and treatment. Statins, ezetimibe, proprotein convertase subtilisin/kexin type 9 (PCSK9) inhibitors, and related strategies reduce arterial lipid retention, modified lipoprotein uptake, and foam cell formation [4,5,6,7,82,83]. However, lipid lowering may not fully reverse advanced plaque pathology once senescence-like foam cells, mitochondrial redox failure, ferroptosis-prone macrophages, defective efferocytosis, and necrotic cores are established. In such lesions, additional strategies may be required to target foam cell fate failure. Senescence and SASP modulation represent one therapeutic entry point. Senescence-like foam cells can persist as inflammatory hubs within advanced plaques, and their SASP-related mediators may amplify endothelial activation, monocyte recruitment, vascular smooth muscle cell dysfunction, extracellular matrix degradation, and fibrous cap weakening [18,21,22,23,24,25,26,27,45,84,85,86]. Conceptually, senomorphic approaches may offer an advantage in plaques with impaired efferocytosis by limiting SASP-related signaling without abruptly increasing the burden of cellular debris; however, this possibility has not been directly tested in advanced atherosclerotic plaques [25,26,87,88].

The therapeutic approaches discussed below have different levels of supporting evidence. Lipid-lowering therapy is clinically established. Senomorphic therapy, mitochondrial redox restoration, ferroptosis-targeted intervention, and pro-efferocytic therapy are largely preclinical or conceptual in the context of advanced plaque non-resolution. We therefore classify proposed interventions by translational readiness rather than implying equivalent validation.

The lack of cardiovascular benefit in a large randomized trial of combined antioxidant vitamin supplementation highlights the limitations of nonspecific systemic antioxidant approaches [89]. Importantly, such supplementation was not designed to selectively restore mitochondrial NADPH-dependent redox buffering, glutathione/thioredoxin recycling, GPX4-dependent phospholipid hydroperoxide detoxification, mitophagy, or efferocytosis within advanced plaques. Future redox-based therapy should therefore be plaque-stage, cell-lineage, and compartment specific rather than broadly antioxidant.

**Figure 3:**
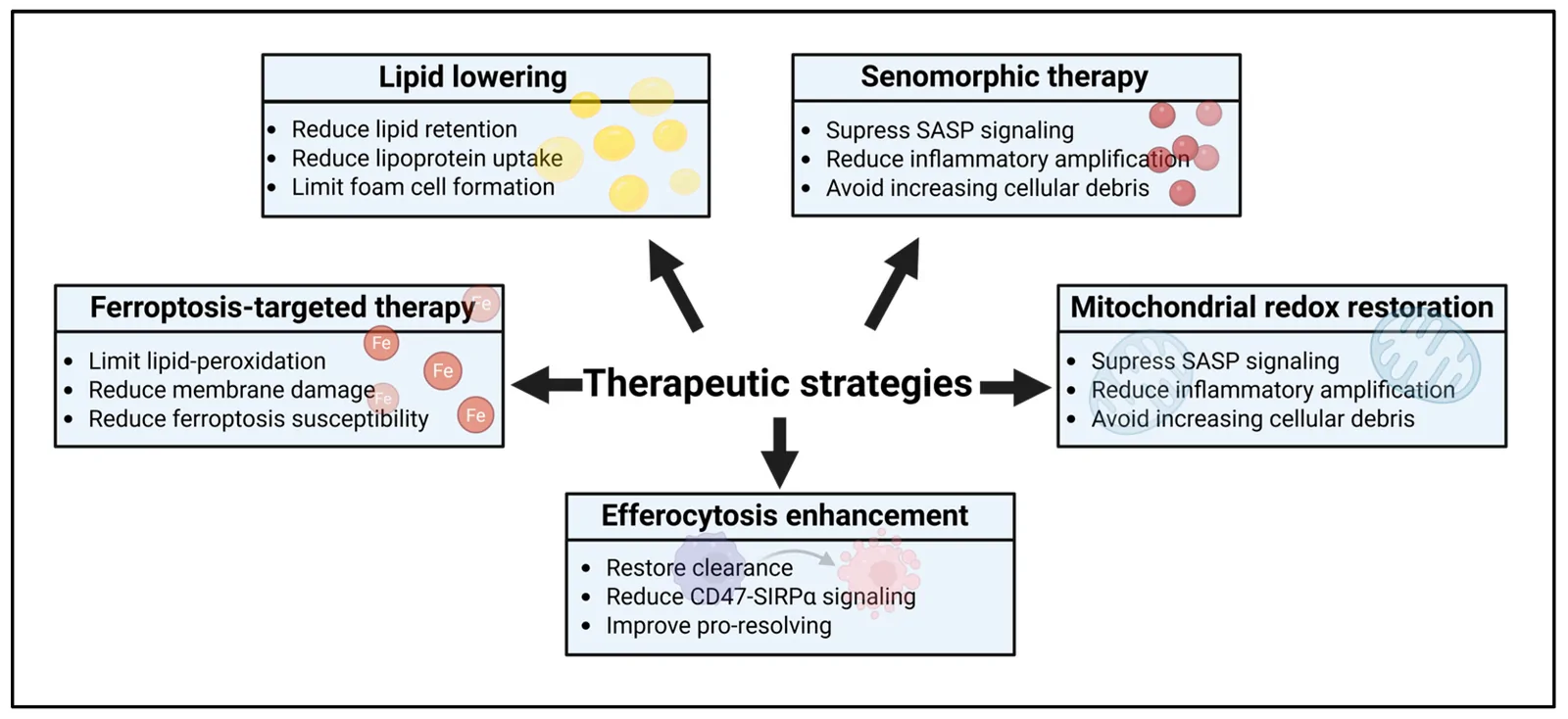
Therapeutic strategies targeting mitochondrial redox failure-driven plaque non-resolution. The proposed model identifies several therapeutic entry points for advanced atherosclerosis. Lipid-lowering therapy remains foundational by reducing the upstream burden of apoB-containing lipoproteins and modified lipid uptake. Senomorphic strategies may suppress maladaptive SASP signaling and limit inflammatory amplification without directly increasing cellular debris. Mitochondrial redox restoration may preserve NADPH-dependent antioxidant buffering, glutathione and thioredoxin recycling, GPX4 activity, NRF2-related cytoprotection, and mitophagy. Ferroptosis-targeted strategies may limit lipid peroxide-driven membrane injury by supporting GPX4 function, preserving glutathione availability, reducing redox-active iron, modulating oxidizable phospholipid remodeling, or trapping lipid radicals. Pro-efferocytic strategies may restore MerTK/LRP1-dependent clearance, reduce excessive CD47-SIRPα signaling, improve macrophage metabolic fitness, and promote resolution. Plaque-targeted delivery systems may improve therapeutic specificity; current preclinical proof-of-concept is strongest for pro-resolving and pro-efferocytic approaches, whereas targeted delivery of redox, ferroptosis, or senomorphic agents remains conceptual. Evidence levels are indicated as clinical, preclinical, or conceptual to avoid implying that all therapeutic strategies have equivalent translational readiness. Created in BioRender. Kim, C. S. (2026) https://BioRender.com/yy63xry.

Mitochondrial redox restoration directly targets the proposed transition point between senescence-like persistence and ferroptosis susceptibility. The goal is not to eliminate all reactive oxygen species, because redox signaling is required for normal macrophage function. Rather, the goal is to preserve redox balance sufficiently to prevent senescence-like foam cells from crossing the ferroptotic threshold. Potential targets include mitochondrial ROS generation, NADPH-dependent antioxidant buffering, glutathione and thioredoxin recycling, GPX4 activity, nuclear factor erythroid 2-related factor 2 (NRF2)-related cytoprotection, mitochondrial dynamics, and mitophagy [28,29,30,34,46,47,48,49,51,52,53,54,55,56,57,58,59,60,67,68,81]. Ferroptosis-targeted therapy addresses a downstream consequence of redox failure. Potential strategies include preserving GPX4 activity, supporting glutathione availability, limiting redox-active iron, modulating ACSL4-dependent oxidizable phospholipid remodeling, and trapping lipid radicals [31,32,33,35,36,61,62,63,64,65,70]. These approaches may reduce lipid peroxide-driven membrane damage and limit the generation of oxidized debris. Because ferroptosis-related pathways are important in multiple tissues, plaque-selective or macrophage-targeted delivery may be required to improve safety and specificity. Efferocytosis enhancement is also relevant once advanced plaques contain dying cells and necrotic debris. Therapeutic strategies may aim to preserve MerTK signaling, support LRP1-mediated clearance, reduce excessive CD47-SIRPα signaling, improve macrophage metabolic fitness, or activate specialized pro-resolving mediator pathways [37,38,39,40,41,42,43,78,79]. Preclinical studies demonstrate the feasibility of plaque- or lesional-macrophage-targeted delivery of pro-resolving and pro-efferocytic therapies [90,91]. Extension of these delivery platforms to senomorphic agents, mitochondrial redox modulators, or ferroptosis-targeted drugs remains a translational concept that requires direct testing. Therapeutic strategies can be organized according to the major failure points in this pathway: SASP-driven inflammatory persistence, mitochondrial redox collapse, ferroptotic lipid peroxidation, defective efferocytosis, and plaque non-resolution (Figure 3). The major checkpoints and therapeutic implications of this model are summarized in Table 1.

## 5. Future Perspectives and Conclusions

The proposed model that mitochondrial redox failure links senescence-like foam cell stress to ferroptosis and plaque non-resolution provides a focused framework for understanding advanced atherosclerosis. However, this model should be considered a testable hypothesis rather than a fully established linear pathway. Future studies need to determine whether senescence-like foam cells directly acquire ferroptosis-prone features in human plaques, which mitochondrial redox checkpoints regulate this transition, and how defective efferocytosis converts foam cell injury into necrotic core expansion and plaque instability. A major priority is to define senescence-like and ferroptosis-prone foam cells more precisely in human atherosclerotic lesions. Single-cell studies have revealed substantial heterogeneity among plaque macrophages [17,20,72,73,74], but the relationship among lipid loading, senescence-like stress states, mitochondrial redox dysfunction, lipid peroxidation, and defective clearance remains incompletely resolved. Future studies should integrate single-cell transcriptomics, spatial transcriptomics, lipidomics, redox imaging, mitochondrial functional assays, and histological validation to identify foam cell states that meet composite criteria for a senescence-like phenotype, ferroptosis susceptibility, or defective efferocytosis.

Marker selection will be critical. Senescence-like foam cell stress should not be inferred from a single marker such as p16, p21, or senescence-associated beta-galactosidase activity. Instead, senescence-like foam cell states should be defined by combined evidence of DNA damage signaling, mitochondrial dysfunction, SASP production, impaired stress adaptation, and defective resolution features [21,22,23,24,25,26,27]. Similarly, ferroptosis susceptibility should be evaluated using lipid peroxide accumulation, GPX4 functional capacity, glutathione status, ACSL4-related lipid remodeling, iron accumulation, and rescue by ferroptosis-targeted interventions [31,32,33,35,36,61,62,63,64,65,70]. Spatial co-localization of these features near necrotic cores, hypoxic regions, intraplaque hemorrhage, or macrophage-rich shoulder areas may help identify plaque niches where the proposed transition is most active. A second priority is to establish causality. Advanced plaques contain overlapping forms of cell stress and death, including apoptosis, necroptosis, pyroptosis, secondary necrosis, and ferroptosis. Therefore, the coexistence of senescence markers and ferroptosis-related markers does not prove that senescence-like foam cells directly progress toward ferroptotic injury. Causal validation will require lineage tracing, inducible macrophage-specific models, mitochondrial redox reporter systems, lipid peroxide imaging, ferroptosis rescue experiments, and temporal single-cell trajectory analysis. These approaches may clarify whether mitochondrial redox failure is a driver of foam cell fate transition or a marker of advanced cellular injury [17,21,24,30,31,32,37,51,72,73,74,75]. The interaction between ferroptosis and efferocytosis also requires deeper investigation. Most efferocytosis studies focus on apoptotic cell clearance, but lipid-peroxidized or ferroptotic cells may generate distinct oxidized lipid signals, membrane alterations, and inflammatory debris. Whether ferroptotic foam cells are efficiently recognized and cleared by plaque macrophages remains unclear. Future studies should examine how MerTK, LRP1, CD47-SIRPα signaling, macrophage metabolic fitness, lysosomal capacity, and pro-resolving mediator pathways respond to ferroptotic foam cell debris [37,38,39,40,41,42,43,78,79]. From a translational perspective, this framework suggests that advanced atherosclerosis should be stratified by plaque biology rather than treated only according to systemic lipid burden. Early lesions may benefit primarily from prevention of foam cell formation, whereas advanced plaques may require restoration of redox resilience, suppression of maladaptive SASP signaling, limitation of ferroptotic lipid peroxidation, and enhancement of efferocytosis-mediated resolution [79,82,83,87,88,90,91]. Biomarkers such as oxidized phospholipids, lipid peroxide signatures, inflammatory mediators, extracellular vesicle cargo, plaque inflammation imaging, iron-sensitive imaging, and necrotic core imaging may help identify patients most likely to benefit from these strategies.

For clinical translation, tissue-based spatial profiling should be complemented by non-invasive or minimally invasive strategies. Circulating extracellular vesicles may provide plaque-related information because they carry cell-derived proteins, nucleic acids, and lipids, although EV isolation, cell-of-origin assignment, and cargo validation require standardization [92]. Imaging approaches may also provide indirect readouts of plaque biology. 18F-FDG PET can assess vascular inflammation, 18F-NaF PET can detect active microcalcification, and somatostatin receptor-targeted tracers such as 68Ga-DOTATATE have been explored for macrophage-rich inflammatory plaques [93,94]. These modalities are not ferroptosis-specific, but they may help stratify plaque activity and identify patients who could benefit from targeted redox- or resolution-based interventions.

In conclusion, foam cells should not be viewed only as lipid-laden macrophages generated by modified lipoprotein uptake and insufficient cholesterol efflux. In advanced plaques, foam cells may become senescence-like inflammatory cells that persist under chronic lipid, oxidative, inflammatory, and mitochondrial stress. Mitochondrial redox buffering may determine whether these cells remain viable or become vulnerable to ferroptotic lipid peroxide injury. When NADPH-dependent antioxidant recycling, glutathione and thioredoxin systems, mitophagy, and GPX4-mediated lipid peroxide detoxification fail, senescence-like foam cells may cross a redox threshold and become ferroptosis-prone. The pathological impact of this transition depends on efferocytosis. If lipid-peroxidized or dying foam cells are efficiently cleared, tissue injury may be limited. Defective clearance leads to an accumulation of oxidized lipids, debris, mitochondrial components, and inflammatory mediators, driving secondary necrosis, core expansion, and plaque instability. Thus, mitochondrial redox failure provides a plausible mechanistic bridge between senescence-like foam cell stress, ferroptosis susceptibility, and failed plaque resolution. This focused framework shifts the review from a broad discussion of foam cell biology to a defined mechanism of foam cell fate failure. Therapeutically, advanced plaque stabilization may require combined strategies that reduce lipid burden, preserve mitochondrial redox resilience, suppress maladaptive SASP signaling, limit ferroptotic lipid peroxidation, enhance efferocytosis, and promote tissue resolution. Defining and targeting this transition may help explain residual cardiovascular risk and support the development of precision therapies for advanced atherosclerosis.

## Figures and Tables

**Table 1:** Key checkpoints linking senescence-like foam cells, mitochondrial redox failure, ferroptosis, and plaque non-resolution.

Checkpoint	Key Mechanisms	Evidence Level	Therapeutic Implication	References
Lineage-diverse foam cell states	Macrophage- and VSMC-derived foam cells; lipid loading; phenotype switching	Strong preclinical lineage-tracing + human observational evidence	Interpret redox-threshold model in a lineage-aware manner	[15,16,17,18,19]
Senescence-like foam cell stress	SASP, p16/p21 or p53 signaling, DNA damage, mitochondrial stress	Preclinical; limited direct human validation	Senomorphic modulation; avoid equating with M1 polarization	[21,22,23,24,25,26,27]
Mitochondrial redox failure	mtROS accumulation, NADPH depletion, GSH/Trx impairment	Mechanistic/preclinical	Mitochondrial redox restoration	[28,29,30,54,55,56,57,58,59,60]
GPX4 functional failure	Reduced lipid peroxide detoxification; lipid hydroperoxide accumulation	Mechanistic/preclinical	GPX4/GSH support	[31,32,33,34,35,36,58,59,60,61,62]
Ferroptosis susceptibility	ACSL4, PUFA-phospholipids, labile iron, lipid radical propagation	Preclinical; limited direct human plaque validation	Ferroptosis-targeted intervention	[31,32,33,34,35,36,61,62,63,64,65,66,67,68,69,70,71]
Defective efferocytosis	MerTK/LRP1 dysfunction; CD47-SIRPα signaling; post-engulfment redox handling (proposed)	Strong preclinical for efferocytosis; post-engulfment component conceptual	Pro-efferocytic therapy	[37,38,39,40,41,42,43,78,79,80]
Plaque non-resolution	Uncleared debris, oxidized lipids, chronic inflammation, necrotic core expansion	Established plaque biology	Combination strategy	[37,78,79]

## Data Availability

No new data were created or analyzed in this study.

## Abbreviations

The following abbreviations are used in this manuscript:

SASP	Senescence-associated secretory phenotype
ROS	Reactive oxygen species
NADPH	Nicotinamide adenine dinucleotide phosphate
GPX4	Glutathione peroxidase 4
DNA	Deoxyribonucleic Acid
MerTK	Myeloid-epithelial-reproductive tyrosine kinase
LRP1	Low-density lipoprotein receptor
CD47	Cluster of Differentiation 47
SIRPα	Signal regulatory protein α
p16	Cyclin-dependent kinase inhibitor 2A
p21	Cyclin-dependent kinase inhibitor 1A
IL-1β	Interleukin-1 beta
IL-6	Interleukin-6
TNF-α	Tumor necrosis factor-alpha
CCL2	Chemokine C-C motif ligand 2
CXCL	Chemokine C-X-C motif ligands
MMPs	Matrix metalloproteinases
NF-κB	Nuclear factor kappa-light-chain-enhancer of activated B cells
ACSL4	Acyl-CoA synthetase long-chain family member 4
PCSK9	Proprotein convertase subtilisin/kexin type 9
NRF2	Nuclear factor erythroid 2-related factor 2
GSH	Reduced glutathione
PUFA	Polyunsaturated fatty acid
VSMC	Vascular smooth muscle cell
mtROS	Mitochondrial reactive oxygen species
4-HNE	4-hydroxynonenal
MDA	Malondialdehyde
FSP1	Ferroptosis suppressor protein 1
EV	Extracellular vesicle
PET	Positron emission tomography
